# Spatial Analyses of Crisis Pregnancy Centers and Abortion Facilities in the United States, 2021 (Pre-Dobbs): Cross-Sectional Study

**DOI:** 10.2196/60001

**Published:** 2024-11-06

**Authors:** Andrea Swartzendruber, Nicole Luisi, Erin R Johnson, Danielle N Lambert

**Affiliations:** 1 Department of Epidemiology and Biostatistics College of Public Health University of Georgia Athens, GA United States

**Keywords:** crisis pregnancy center, abortion, induced, reproductive health, policy, access to information, internet, directory, geographic information system, spatial analyses

## Abstract

**Background:**

Crisis pregnancy centers (CPCs) are religious nonprofit organizations with a primary mission of diverting people from having abortions. One CPC tactic has been to locate near abortion facilities. Despite medical groups’ warnings that CPCs do not adhere to medical and ethical standards and pose risks, government support for CPCs has significantly increased.

**Objective:**

This study aims to map CPCs, abortion facilities, and geographical areas in the United States into 4 zones based on their proximity to CPCs and abortion facilities. We sought to describe the number and percentage of reproductive-aged women living in each zone and the proximity of CPCs to abortion facilities.

**Methods:**

Using 2021 data from CPC Map and the Advancing New Standards in Reproductive Health Abortion Facility Database, we determined the ratio of CPCs to abortion facilities. Along with census data, we categorized and mapped US block groups into 4 distinct zones based on locations of block group centroids within 15-mile (1 mile is approximately 1.609 km) radii of CPCs and abortion facilities, namely “no presence,” “CPC only,” “abortion facility only,” and “dual presence.” We calculated the number and percentage of block groups and reproductive-aged (15-49 years) women living in each zone. We calculated driving distances and drive times from abortion facilities to the nearest CPC and mapped abortion facilities with CPCs in close proximity. All analyses were conducted nationally and by region, division, and state.

**Results:**

Nationally, the ratio of CPCs to abortion facilities was 3.4, and 54.9% (131,410/239,462) of block groups were categorized in the “dual presence” zone, 26.6% (63,679/239,462) as “CPC only,” and 0.8% (63,679/239,462) as “abortion facility only.” Most reproductive-aged women (45,150,110/75,582,028, 59.7%) lived in a “dual presence” zone, 26.1% (19,696,572/75,582,028) in a “CPC only” zone, and 0.8% (625,403/75,582,028) in an “abortion facility only” zone. The number of block groups and women classified as living in each zone varied by region, division, and state. Nationally, the median distance from abortion facilities to the nearest CPC was 2 miles, and the median drive time was 5.5 minutes. Minimum drive times were <1 minute in all but 11 states. The percentages of abortion facilities with a CPC within 0.25, 0.5, 1, and 3 miles were 14.1% (107/757), 22.6% (171/757), 36.1% (273/757), and 66.3% (502/757), respectively.

**Conclusions:**

The findings suggest that CPCs’ tactic of locating near abortion facilities was largely realized before the 2022 US Supreme Court decision that overturned the federal right to abortion. Research on CPCs’ locations and tactics should continue given the dynamic abortion policy landscape and risks posed by CPCs. Tailored programming to raise awareness about CPCs and help people identify and access safe sources of health care may mitigate harm. Increased regulation of CPCs and government divestment may also mitigate CPC harms.

## Introduction

Crisis pregnancy centers (CPCs) are grassroots organizations within the antiabortion movement that hold themselves out as providing “alternatives to abortion” [1-3]. The centers are religious nonprofit organizations that frequently mimic medical clinics and even abortion facilities to reach their antiabortion, anti-contraception, anti–comprehensive sex education, and evangelical goals [4-6]. CPCs particularly target young people, people of color, and people living in low-income households [5,6]. These communities disproportionately experience barriers to health care, are disproportionately burdened by unintended pregnancy and other adverse sexual and reproductive health outcomes, and experience among the highest abortion rates [7-9]. Although, increasingly, CPCs provide limited medical services, they are not medical centers and are not regulated as such [5,6,10]. They frequently provide inaccurate health information in support of their goals and do not adhere to national medical and ethical practice standards [5,6,10]. Major public health and medicine organizations warn that CPCs pose risks to individual, family, and public health [5].

CPCs risk harm by prioritizing their own goals over client needs, failing to adhere to standard medical and ethical practices, failing to promote informed consent, using deceptive advertising, and enacting stigma [5,6,11]. Evidence also shows that CPCs delay abortion care, which risks individual health [12]. However, government funding and support for CPCs increased substantially in the decade before and in the years since the *Dobbs v Jackson Women’s Health* *Organization* decision, which overturned *Roe v Wade* and the federal right to abortion in the United States [13,14].

Over the decades and across the United States, CPCs and their affiliate organizations have made clear their goals to “compete” with abortion facilities [1,15]. Opening and locating near abortion facilities to attract people considering and seeking abortion has been a key strategy encouraged by the umbrella organizations with which CPCs affiliate, such as Heartbeat International (formally called “Alternatives to Abortion International”), at least since the 1990s [1]. In the early 1990s, abortion facilities were primarily located in large cities, and CPCs mainly operated in midsize cities, towns, and rural areas [1]. Abortion facilities continue to be concentrated in urban areas in the United States [16]. Although there have been many anecdotal reports of CPCs locating near abortion facilities to engage with and unwittingly attract people seeking abortion care, to date, a dearth of studies has directly examined the locations of CPCs around abortion facilities.

CPCs operated in every state before the *Dobbs* decision [4]. Many of the states that have banned or severely restricted abortion since the *Dobbs* decision have continued to fund or increased funding for CPCs (eg, Alabama, Florida, Indiana, Iowa, Louisiana, Mississippi, Tennessee, and West Virginia) [14,17-26], whereas other states where abortion remains legal or that have moved to protect abortion access have sought to regulate CPCs, have issued consumer warning alerts, or defunded CPCs (eg, California, Colorado, Illinois, Massachusetts, New Jersey, and Pennsylvania) [27-38].

Studies show that closer proximity to abortion facilities is associated with increased abortion rates [39]. Similarly, proximity to CPCs is associated with an increased likelihood of visiting CPCs for services [12]. Understanding CPC locations and to what extent, how close, and where CPCs were located to abortion facilities before the *Dobbs* decision may serve as a useful baseline for evaluating CPCs’ impact and identifying CPCs’ strategies and tactics after the *Dobbs* decision and may provide a more comprehensive understanding of the extent to which CPCs integrated within the geographic landscape of reproductive health services. Better understanding about where CPCs locate relative to abortion facilities may also identify areas where tailored programming is needed to increase awareness about CPCs, including their objectives and tactics, among reproductive-aged people, particularly communities that CPCs target. Examining relative locations of CPCs and abortion facilities may also be useful for investigating and explaining sexual and reproductive health outcomes.

**Objective**

This study aimed to describe the location of CPCs in the United States relative to abortion facilities in 2021, the year before the *Dobbs* decision. Specifically, we mapped 4 distinct geographic zones based on their location within 15-mile (1 mile is approximately 1.609 km) radii of CPCs and abortion facilities, where (1) neither CPCs and abortion facilities operated, (2) only CPCs operated, (3) only abortion facilities operated, and (4) both CPCs and abortion facilities operated. This study also aimed to describe the number and proportion of women of reproductive age who resided in each zone and to examine distances and drive times from abortion facilities to the nearest CPC and the number and percentage of abortion facilities for which the nearest CPC was located within 0.25, 0.5, 1, and 3 driving miles.

## Methods

### Data Sources

Data about the locations of CPCs in 2021 were obtained from CPC Map [40], a web-based geocoded directory of all the CPCs operating in the United States [4]. CPC Map identifies the brick-and-mortar locations of CPCs that provide free pregnancy testing. It excludes mobile CPC vans, adoption agencies, maternity homes, thrift stores, and offices that are affiliated with CPCs but do not provide free pregnancy testing [4]. CPC Map was launched in 2018 and a major update was released in August 2021, just 10 months before the *Dobbs* decision. To create the 2021 data set, we reviewed all the websites for CPCs included in the 2018 data set to confirm address and contact information, identify new centers, identify centers that were no longer operating, and confirm services offered. We also used systematic internet searches to identify potential new centers and assessed eligibility. We called centers that did not have accessible websites and those with incomplete information on their websites. In 2021, 2546 CPCs were included in the CPC Map database.

Data about the locations of abortion facilities in 2021 were obtained from Advancing New Standards in Reproductive Health’s (ANSIRH’s) Abortion Facility Database [41]. The ANSIRH database provides location information for all abortion facilities in the United States that publicly advertise abortion services on the internet and includes facilities that provided abortion care at any time during the year [41]. The database was developed using systematic internet searches and calls to facilities with incomplete or unclear information [42]. For the current analysis, we included brick-and-mortar facilities that were open and active. We excluded abortion facilities that exclusively offered telehealth services and a single facility without address information. A total of 757 abortion facilities were included in the current analysis.

Census block groups were established based on 2021 cartographic boundaries published by the US Census Bureau [43]. Data about the number of women of reproductive age were obtained from 2020 US Census Demographic and Housing Characteristics [44]. We selected block group as the unit of analysis because US census data are available by both age and sex and practicalities of producing national estimates.

### Ethical Considerations

Both CPC Map and ANSIRH Abortion Facility database were developed using publicly available data. The authors developed and maintain CPC Map and received approval to access the abortion data. Since all CPCs and abortion facilities are deidentified and the research involved no interaction with or information about human participants, this study was not submitted for Institutional Review Board approval per the policy of the Human Research Protection Program (subsections 2.2 and 3.6) of the University of Georgia [45].

### Statistical Analysis

First, we calculated the ratio of abortion facilities nationally and by region, division, and state and mapped the geolocations of CPCs and abortion facilities [46]. Next, using ArcGIS Online (Esri), we generated a 15-mile driving distance buffer zone for each CPC and abortion facility using the Generate Travel Areas tool with default settings for traffic and time of day. Then, we identified the geometric center of each census block group using the Find Centroids tool and assigned entire block groups to distinct zones based on block group centroids’ locations relative to each buffer zone.

We categorized block groups into 1 of 4 zones based on driving distances from CPCs and abortion facilities to block group centroids. Block groups outside a 15-mile driving distance radius of a CPC or abortion facility were categorized as being in a “no presence” zone. Block groups within a 15-mile radius of an abortion facility only were categorized as being in an “abortion facility only” zone. Block groups within a 15-mile radius of a CPC only were categorized as being in a “CPC only” zone. Block groups that were within a 15-mile radius of a CPC and abortion facility were categorized as being in a “dual presence” zone.

We defined zones using a 15-mile driving radius based on prior research. A 2017 study that used county-level analyses reported that the median distance to the nearest abortion facility for reproductive-aged women in the United States was approximately 11 miles in 2014 [16]. County-level analyses have since been shown to result in relatively large underestimates of abortion access [47]. Another published study that used data from the 2014 Abortion Patient Survey reported that patients who obtained abortions services traveled a median of 15.7 miles to an abortion facility [48]. We opted for larger (15 miles) rather than smaller (11 miles) zones given the large difference in the number of CPCs compared with abortion facilities. We expected a smaller zone to yield more extreme results solely based on the relative number of CPCs and abortion facilities.

We used summary statistics to examine the number and percentage of women of reproductive age (15-49 years) living in each zone and the number and percentage of abortion facilities for which the nearest CPC was located within 0.25, 0.5, 1, and 3 driving miles. Spatial analyses were conducted with ArcGIS Online (Esri). Other descriptive statistics were prepared with R Statistical Software (version 4.3.1; R Core Team 2023). All analyses were conducted nationally and by region, division, and state.

## Results

### Ratio of CPCs to Abortion Facilities

**Figure 1:**
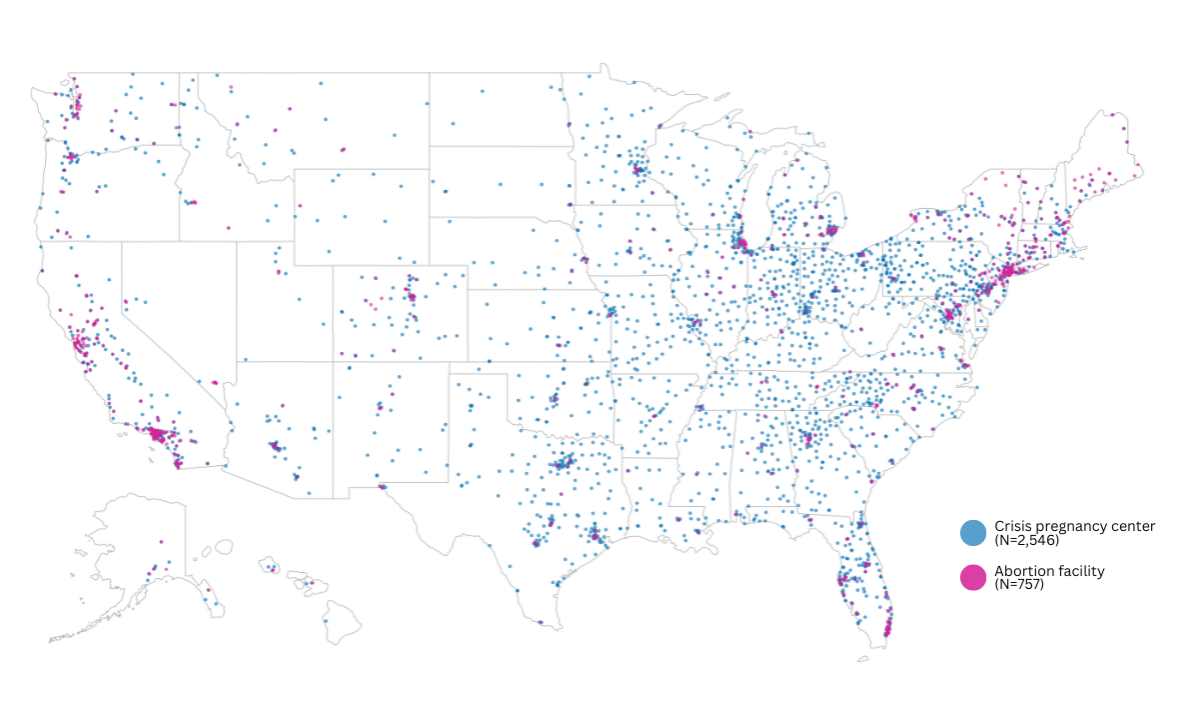
Crisis pregnancy centers (CPCs) and brick-and-mortar abortion facilities in the United States, 2021. Data sources: CPC Map; Advancing New Standards in Reproductive Health Abortion Facility Database.

Multiple CPCs and at least 1 abortion facility were operating in every state in 2021 (Figure 1). Nationally, the ratio of CPCs to abortion facilities in 2021 was 3.4 (Table 1). By region, the ratio was lowest in the West and highest in the Midwest. By division, the ratio ranged from 1.2 in 2 divisions to >10.0 in 3. In only 3 states and the District of Columbia (DC) was the ratio of CPCs to abortion facilities <1. The ratio was 1.0 in a single state and >15.0 in 6.

**Table 1:** Number of crisis pregnancy centers (CPCs) and ratio of CPCs to brick-and-mortar abortion facilities, in the United States and by region, division, and state, 2021.

		Number of CPCs	Ratio of CPCs to abortion facilities
**United States**		2546	3.4
**Region**			
	Midwest	745	7.6
	Northeast	375	1.8
	South	994	5.7
	West	432	1.6
**Division**			
	East North Central	480	6.4
	East South Central	196	13.1
	Middle Atlantic	291	2.0
	Mountain	185	3.1
	New England	84	1.2
	Pacific	247	1.2
	South Atlantic	481	3.7
	West North Central	265	11.5
	West South Central	317	10.6
**State**			
	Alabama	52	10.4
	Alaska	10	2.0
	Arizona	47	5.9
	Arkansas	40	20.0
	California	148	0.9
	Colorado	51	2.1
	Connecticut	20	1.3
	Delaware	5	2.5
	District of Columbia	2	0.5
	Florida	151	2.7
	Georgia	88	6.3
	Hawaii	6	2.0
	Idaho	19	4.8
	Illinois	97	3.6
	Indiana	96	13.7
	Iowa	43	7.2
	Kansas	38	9.5
	Kentucky	55	27.5
	Louisiana	32	10.7
	Maine	11	0.6
	Maryland	40	2.0
	Massachusetts	29	1.6
	Michigan	105	3.8
	Minnesota	73	10.4
	Mississippi	29	29.0
	Missouri	73	73.0
	Montana	17	2.8
	Nebraska	20	6.7
	Nevada	7	0.8
	New Hampshire	14	2.3
	New Jersey	39	1.0
	New Mexico	24	4.8
	New York	92	1.1
	North Carolina	89	5.6
	North Dakota	7	7.0
	Ohio	124	13.8
	Oklahoma	47	15.7
	Oregon	38	2.9
	Pennsylvania	160	10.0
	Rhode Island	3	1.5
	South Carolina	35	11.7
	South Dakota	11	11.0
	Tennessee	60	8.6
	Texas	198	9.0
	Utah	8	4.0
	Vermont	7	1.2
	Virginia	54	3.6
	Washington	45	1.6
	West Virginia	17	17.0
	Wisconsin	58	14.5
	Wyoming	12	12.0

### Block Groups by Zone

**Figure 2:**
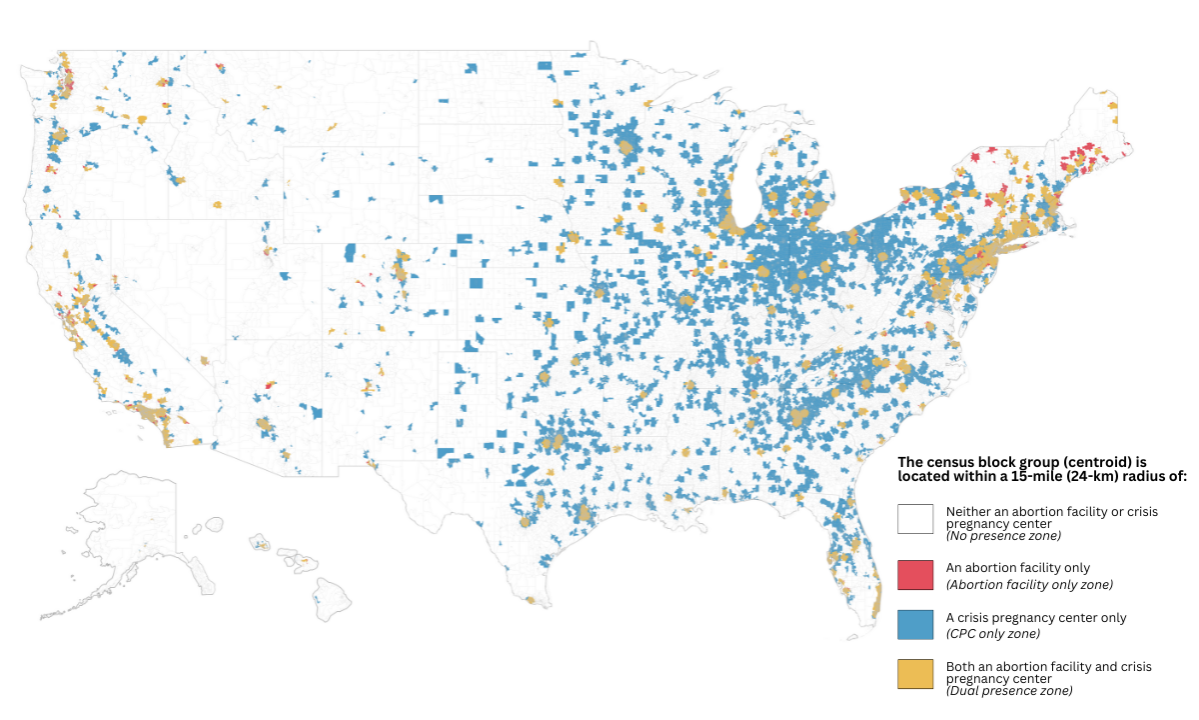
United States Census block groups within crisis pregnancy center (CPC) and abortion facility presence zones, 2021. Data sources: CPC Map; Advancing New Standards in Reproductive Health Abortion Facility Database.

We mapped block groups by zone (Figure 2) and determined the number and percentage of block groups categorized into each zone nationally and by region, division, and state (Table 2). More than half of block groups (131,410/239,462, 54.9%) in the United States were within 15 miles of both a CPC and an abortion facility and categorized in the “dual presence” zone. More than one-quarter (63,679/239,462, 26.6%) of block groups were in the “CPC only” zone. Less than one-fifth (42,447/239,462, 17.7%) of block groups were categorized in the “no presence” zone. Less than 1% (1926/239,462) of block groups nationally were categorized in the “abortion facility only” zone.

By region, approximately three-fourths of block groups in the Northeast and West were categorized in the “dual presence” zone, whereas less than half in the Midwest and South were categorized in the “dual presence” zone. More than one-third of block groups in the Midwest and South were categorized in the “CPC only” zone as compared with 15.8% in the Northeast and 11.9% in the West. The South had the highest and the Northeast the lowest percentage of block groups categorized into the “no presence” zone. The Northeast and West had >1% of block groups categorized in the “abortion facility only” zone, whereas the percentage was 0.1% in both the South and Midwest.

By division, the percentage of block groups categorized in the “dual presence” zone ranged from 27.2% to 78.9%. The percentage was <50% in only 3 divisions. Block groups categorized as “CPC only” ranged from 7.5% to more than one-third in 4 divisions. Block groups categorized in the “no presence” zone ranged from 7.9% to approximately one-third in 2 divisions. The percentage categorized as “abortion facility only” was <1% in most divisions.

In only 2 states was the percentage of block groups in the “dual presence” zone <10%; the percentage was >75% in 6 states and DC. The percentage categorized as “CPC only” ranged from ≤5% in 2 states and DC to >50% in 4 states. The percentage assigned to the “no presence” zone ranged from <10% in DC and 7 states to >50% in 4 states. The percentage categorized in the “abortion facility only” zone was <1% in 42 states and DC.

**Table 2:** Number and percentage of block groups in the United States categorized by their location within a 15-mile (24-km) radius of a crisis pregnancy center (CPC) and abortion facility, 2021.

		Block groups outside of a 15-mile radius of both a CPC and an abortion facility (no presence zone), n (%)	Block groups within a 15-mile radius of an abortion facility only (abortion facility–only zone), n (%)	Block groups within a 15-mile radius of a CPC only (CPC-only zone), n (%)	Block groups within a 15-mile radius of both a CPC and abortion facility (dual presence zone), n (%)
**United States**		42,447 (17.7)	1,926 (0.8)	63,679 (26.6)	131,410 (54.9)
**Region**					
	Midwest	10,453 (18.8)	51 (0.1)	19,911 (35.8)	25,129 (45.2)
	Northeast	4423 (10)	670 (1.5)	6985 (15.8)	32,037 (72.6)
	South	19,352 (22.2)	112 (0.1)	30,551 (35)	37,302 (42.7)
	West	8219 (15.7)	1093 (2.1)	6323 (11.9)	36,942 (70.4)
**Division**					
	East North Central	4992 (13.3)	41 (0.1)	13,735 (36.5)	18,904 (50.2)
	East South Central	4566 (31.5)	31 (0.2)	5971 (41.2)	3942 (27.2)
	Middle Atlantic	2579 (7.9)	377 (1.2)	5325 (16.2)	24,493 (74.7)
	Mountain	4286 (25.1)	210 (1.2)	3566 (20.9)	9007 (52.8)
	New England	1844 (16.3)	293 (2.6)	1660 (14.6)	7544 (66.5)
	Pacific	3933 (11.1)	883 (2.5)	2666 (7.5)	27,935 (78.9)
	South Atlantic	7971 (18)	79 (0.2)	13,768 (31.1)	22,409 (50.7)
	West North Central	5461 (30.6)	10 (0.1)	6176 (34.6)	6225 (34.8)
	West South Central	6815 (23.8)	2 (0)	10,812 (37.8)	10,951 (38.3)
**State**					
	Alabama	1149 (29.3)	7 (0.2)	1397 (35.6)	1371 (34.9)
	Alaska	214 (42.5)	1 (0.2)	50 (9.9)	239 (47.4)
	Arizona	979 (20.5)	1 (0)	1039 (21.8)	2754 (57.7)
	Arkansas	864 (37.7)	0 (0)	1157 (50.4)	273 (11.9)
	California	1941 (7.6)	408 (1.6)	1376 (5.4)	21,860 (85.4)
	Colorado	585 (14.4)	27 (0.7)	644 (15.9)	2802 (69)
	Connecticut	172 (6.3)	21 (0.8)	111 (4.1)	2408 (88.8)
	Delaware	126 (17.9)	0 (0)	142 (20.2)	435 (61.9)
	District of Columbia	0 (0)	0 (0)	0 (0)	571 (100)
	Florida	1367 (10.2)	26 (0.2)	3466 (26)	8491 (63.6)
	Georgia	1303 (17.5)	3 (0)	2912 (39.1)	3223 (43.3)
	Hawaii	310 (29.3)	6 (0.6)	197 (18.6)	545 (51.5)
	Idaho	477 (37.1)	1 (0.1)	336 (26.2)	470 (36.6)
	Illinois	1192 (12)	8 (0.1)	2299 (23.2)	6397 (64.6)
	Indiana	620 (11.7)	0 (0)	2697 (51)	1970 (37.3)
	Iowa	1130 (41.8)	7 (0.3)	909 (33.6)	657 (24.3)
	Kansas	712 (28.9)	0 (0)	853 (34.7)	896 (36.4)
	Kentucky	1161 (32.4)	0 (0)	1623 (45.3)	797 (22.3)
	Louisiana	1407 (32.8)	2 (0)	1518 (35.4)	1358 (31.7)
	Maine	436 (37)	154 (13.1)	120 (10.2)	468 (39.7)
	Maryland	315 (7.7)	4 (0.1)	607 (14.9)	3143 (77.2)
	Massachusetts	564 (11)	62 (1.2)	1109 (21.7)	3377 (66.1)
	Michigan	1202 (14.4)	22 (0.3)	2216 (26.6)	4904 (58.8)
	Minnesota	1113 (23.7)	2 (0)	1288 (27.4)	2300 (48.9)
	Mississippi	1157 (47.4)	0 (0)	985 (40.3)	301 (12.3)
	Missouri	1202 (23.9)	1 (0)	2579 (51.3)	1249 (24.8)
	Montana	423 (47)	8 (0.9)	169 (18.8)	300 (33.3)
	Nebraska	521 (31.6)	0 (0)	279 (16.9)	848 (51.5)
	Nevada	283 (14.4)	57 (2.9)	98 (5	1525 (77.7)
	New Hampshire	244 (24.5)	49 (4.9)	199 (20)	504 (50.6)
	New Jersey	270 (4.1)	62 (0.9)	386 (5.9)	5875 (89.1)
	New Mexico	485 (30)	5 (0.3)	571 (35.4)	553 (34.3)
	New York	1429 (8.9)	314 (2)	1077 (6.7)	13,189 (82.4)
	North Carolina	1713 (24.2)	26 (0.4)	2750 (38.8)	2603 (36.7)
	North Dakota	380 (60.1)	0 (0)	129 (20.4)	123 (19.5)
	Ohio	737 (7.8)	11 (0.1)	4483 (47.4)	4236 (44.7)
	Oklahoma	1056 (31.3)	0 (0)	944 (28)	1374 (40.7)
	Oregon	581 (19.6)	28 (0.9)	623 (21)	1731 (58.4)
	Pennsylvania	880 (8.7)	1 (0)	3862 (38)	5429 (53.4)
	Rhode Island	137 (17.3)	0 (0)	69 (8.7)	585 (74)
	South Carolina	1032 (30.3)	4 (0.1)	1465 (43)	906 (26.6)
	South Dakota	403 (58.1)	0 (0)	139 (20)	152 (21.9)
	Tennessee	1099 (24.1)	24 (0.5)	1966 (43.1)	1473 (32.3)
	Texas	3488 (18.7)	0 (0)	7193 (38.6)	7946 (42.7)
	Utah	845 (41.8)	111 (5.5)	469 (23.2)	595 (29.5)
	Vermont	291 (52.7)	7 (1.3)	52 (9.4)	202 (36.6)
	Virginia	1275 (21.4)	16 (0.3)	1767 (29.7)	2897 (48.6)
	Washington	887 (16.7)	440 (8.3)	420 (7.9)	3560 (67.1)
	West Virginia	840 (51.3)	0 (0)	659 (40.2)	140 (8.5)
	Wisconsin	1241 (26.5)	0 (0)	2040 (43.6)	1397 (29.9)
	Wyoming	209 (45.7)	0 (0)	240 (52.5)	8 (1.8)

### Reproductive-Aged Women Living in Each Zone

We calculated the number and proportion of reproductive-aged women categorized as living within each zone in 2021, nationally and by region, division, and state (Table 3). Nationally, most women aged 15 to 49 years (59.7%) lived in the “dual presence” zone, and more than one-quarter lived in a “CPC only” zone. Less than 1 in 7 (14.3%) lived in a “no presence zone” and only 0.8% lived in an “abortion facility only” area.

**Table 3:** Number and percentage of women aged 15 to 49 years in the United States residing within a 15-mile (24-km) radius radius of a crisis pregnancy center (CPC) and abortion facility, 2021.

Location (number of women 15-49 years)			Women who lived outside of a 15-mile radius of both A CPC and an abortion facility (no presence zone), n (%)		Women who lived within a 15-mile radius of an abortion facility only (abortion facility–only zone), n (%)		Women who lived within a 15-mile radius of A CPC only (CPC-only zone), n (%)		Women who lived within a 15-mile radius of both a CPC and abortion facility (dual presence zone), n (%)
**United States**			10,109,943 (13.4)		625,403 (0.8)		19,696,572 (26.1)		45,150,110 (59.7)
**Region**									
	Midwest	2,149,730 (14.1)		15,622 (0.1)		5,474,481 (35.9)		7,618,288 (49.9)	
	Northeast	947,254 (7.2)		171,165 (1.3)		1,864,296 (14.2)		10,101,787 (77.2)	
	South	4,823,254 (16.7)		39,766 (0.1)		10,386,971 (35.9)		13,709,252 (47.3)	
	West	2,189,705 (10)		398,850 (2.2)		1,970,824 (10.8)		13,720,783 (75.1)	
**Division**									
	East North Central	1,044,280 (9.9)		11,670 (0.1)		3,671,594 (34.9)		5,781,054 (55)	
	East South Central	1,129,365 (25.8)		12,411 (0.3)		1,949,048 (44.5)		1,288,215 (29.4)	
	Middle Atlantic	528,894 (5.5)		101,453 (1)		1,364,117 (14.1)		7,690,587 (79.4)	
	Midwest	2,149,730 (14.1)		15,622 (0.1)		5,474,481 (35.9)		7,618,288 (49.9)	
	Mountain	1,168,835 (20.6)		96,521 (1.7)		1,133,533 (20)		3,265,353 (57.6)	
	New England	418,360 (12.3)		69,712 (2.1)		500,179 (14.7)		2,411,200 (70.9)	
	Pacific	1,020,870 (8.1)		302,329 (2.4)		837,291 (6.6)		10,455,430 (82.9)	
	South Atlantic	2,004,962 (13.4)		26,694 (0.2)		4,586,992 (30.7)		8.301,860 (55.6)	
	West North Central	276,073 (21.8)		0 (0)		1,802,887 (38)		1,837,234 (38.7)	
	West South Central	1,688,927 (17.5)		661 (0)		3,850,931 (39.9)		4,119,177 (42.6)	
**State**									
	Alabama	274,439 (24.1)		3413 (0.3)		438,581 (38.5)		422,444 (37.1)	
	Alaska	64,376 (39.1)		340 (0.2)		21,500 (13.1)		78,409 (47.6)	
	Arizona	252,530 (16.1)		84 (0)		324,918 (20.7)		991,101 (63.2)	
	Arkansas	186,313 (27.9)		0 (0)		390,339 (58.4)		91,256 (13.7)	
	California	523,724 (5.6)		140,404 (1.5)		449,947 (4.8)		8,295,997 (88.2)	
	Colorado	148,348 (10.9)		9539 (0.7)		189,189 (13.9)		1,016,400 (74.5)	
	Connecticut	38,058 (4.8)		5213 (0.7)		24,129 (3)		732,584 (91.6)	
	Delaware	22,371 (10.4)		0 (0)		48,440 (22.5)		144,011 (67)	
	District of Columbia	0 (0)		0 (0)		0 (0)		215,054 (100)	
	Florida	371,060 (8.1)		6793 (0.1)		1,124,675 (24.5)		3,087,436 (67.3)	
	Georgia	343,125 (13.3)		1254 (0)		1,021,499 (39.7)		1,209,071 (47)	
	Hawaii	90,187 (29.1)		1765 (0.6)		66,925 (21.6)		150,775 (48.7)	
	Idaho	120,393 (29.6)		203 (0)		117,425 (28.9)		168,308 (41.4)	
	Illinois	227,612 (7.7)		1455 (0)		616,053 (20.9)		2,103,928 (71.3)	
	Indiana	138,446 (9.1)		0 (0)		724,692 (47.8)		651,722 (43)	
	Iowa	219,739 (32)		2786 (0.4)		246,640 (36)		216,656 (31.6)	
	Kansas	142,549 (22.1)		0 (0)		243,669 (37.7)		259,394 (40.2)	
	Kentucky	260,112 (26.1)		0 (0)		503,657 (50.6)		231,357 (23.2)	
	Louisiana	307,130 (28.8)		661 (0.1)		365,725 (34.3)		391,470 (36.8)	
	Maine	84,900 (30.7)		33,073 (12)		33,227 (12)		125,187 (45.3)	
	Maryland	82,860 (5.8)		1106 (0.1)		214,229 (14.9)		1,140,822 (79.3)	
	Massachusetts	136,601 (8.3)		17,087 (1)		356,943 (21.7)		1,136,257 (69)	
	Michigan	233,766 (10.6)		6182 (0.3)		561,663 (25.6)		1,395,184 (63.5)	
	Minnesota	218,902 (17.3)		914 (0.1)		371,688 (29.4)		672,825 (53.2)	
	Mississippi	273,673 (40.9)		0 (0)		296,536 (44.3)		99,264 (14.8)	
	Missouri	243,814 (17.9)		252 (0)		783,798 (57.6)		332,663 (24.5)	
	Montana	82,674 (36.7)		2156 (1)		51,220 (22.7)		89,403 (39.7)	
	Nebraska	103,262 (23.7)		0 (0)		72,719 (16.7)		259,122 (59.6)	
	Nevada	71,733 (10.1)		25,553 (3.6)		26,522 (3.7)		584,440 (82.5)	
	New Hampshire	54,946 (18.9)		12,845 (4.4)		52,418 (18.1)		169,856 (58.6)	
	New Jersey	73,272 (3.5)		18,762 (0.9)		94,911 (4.5)		1,920,326 (91.1)	
	New Mexico	108,859 (23.7)		1002 (0.2)		163,615 (35.6)		185,682 (40.4)	
	New York	270,709 (5.7)		82,432 (1.7)		270,750 (5.7)		4,129,864 (86.9)	
	North Carolina	438,613 (18.4)		10,955 (0.5)		855,600 (35.8)		1,083,256 (45.4)	
	North Dakota	87,715 (51.2)		0 (0)		39,423 (23)		44,340 (25.9)	
	Ohio	168,383 (6.5)		4033 (0.2)		1,203,655 (46.6)		1,206,096 (46.7)	
	Oklahoma	230,365 (26)		0 (0)		266,693 (30.1)		388,664 (43.9)	
	Oregon	119,257 (12.4)		7410 (0.8)		184,859 (19.3)		646,589 (67.5)	
	Pennsylvania	184,913 (6.5)		259 (0)		998,456 (35.4)		1,640,397 (58.1)	
	Rhode Island	42,411 (16.9)		0 (0)		21,861 (8.7)		186,074 (74.3)	
	South Carolina	256,293 (22.7)		2880 (0.3)		500,719 (44.4)		367,944 (32.6)	
	South Dakota	89,469 (47.9)		0 (0.)		44,950 (24.1)		42,234 (28)	
	Tennessee	321,141 (20.4)		8998 (0.6)		710,274 (45.1)		535,150 (34)	
	Texas	965,119 (13.7)		0 (0)		2,828,174 (40.2)		3,247,787 (46.1)	
	Utah	334,264 (41.2)		57,984 (7.2)		190,754 (23.5)		227,594 (28.1)	
	Vermont	61,444 (45.3)		1494 (1.1)		11,601 (8.5)		61,242 (45.1)	
	Virginia	315,680 (15.8)		3706 (0.2)		657,469 (32.9)		1,022,109 (51.1)	
	Washington	223,326 (12.6)		152,410 (8.6)		114,060 (6.4)		1,283,660 (72.4)	
	West Virginia	174,960 (47.1)		0 (0)		164,361 (44.2)		32,167 (8.7)	
	Wisconsin	276,073 (21.8)		0 (0)		565,531 (44.7)		424,124 (33.5)	
	Wyoming	50,034 (40.9)		0 (0)		69,890 (57.1)		2425 (2)	

More than three-quarters of women lived in a “dual presence” zone in the Northeast and West regions, whereas nearly half did in the Midwest and South. More than one-third of women in the Midwest and South lived in a “CPC only” zone; the percentage was <15% in the Northeast and West. The percentage of women who lived in a “no presence” zone ranged from 7.2% (in the Northeast to 16.7% in the South. The percentage of women who lived in an “abortion facility only” zone was 0.1% both in the Midwest and South, 1.3% in the Northeast, and 2.2% in the West.

The percentage of women living in a “dual presence” zone by division ranged from 29.4% to 82.9%. Most women lived in a “dual presence” zone in all but 3 divisions. The percentage of women who lived in a “CPC only” zone ranged from 6.6% to 44.5%. Less than 10% of women of reproductive age lived in a “no presence” zone in 3 divisions and >20% did in 3. The percentage of women who lived in an “abortion facility only” zone ranged from 0 to 2.1%.

By state, the percentage of reproductive-aged women who lived in a “dual presence” zone ranged from 2% to 100%. In 21 states and DC, more than half of women lived in a “dual presence” zone. The percentage who lived in a “CPC only” zone was <5% in 4 states and DC and >50% in 4 states. The percentage who lived in a “no presence” zone ranged from 0% to >45% in 4 states. The percentage who lived in an “abortion facility only” zone ranged from 0% in DC and 21 states to 12%. The percentage was >1% in only 8 states.

### Driving Distance and Drive Times From Abortion Facilities to the Nearest CPC

**Figure 3:**
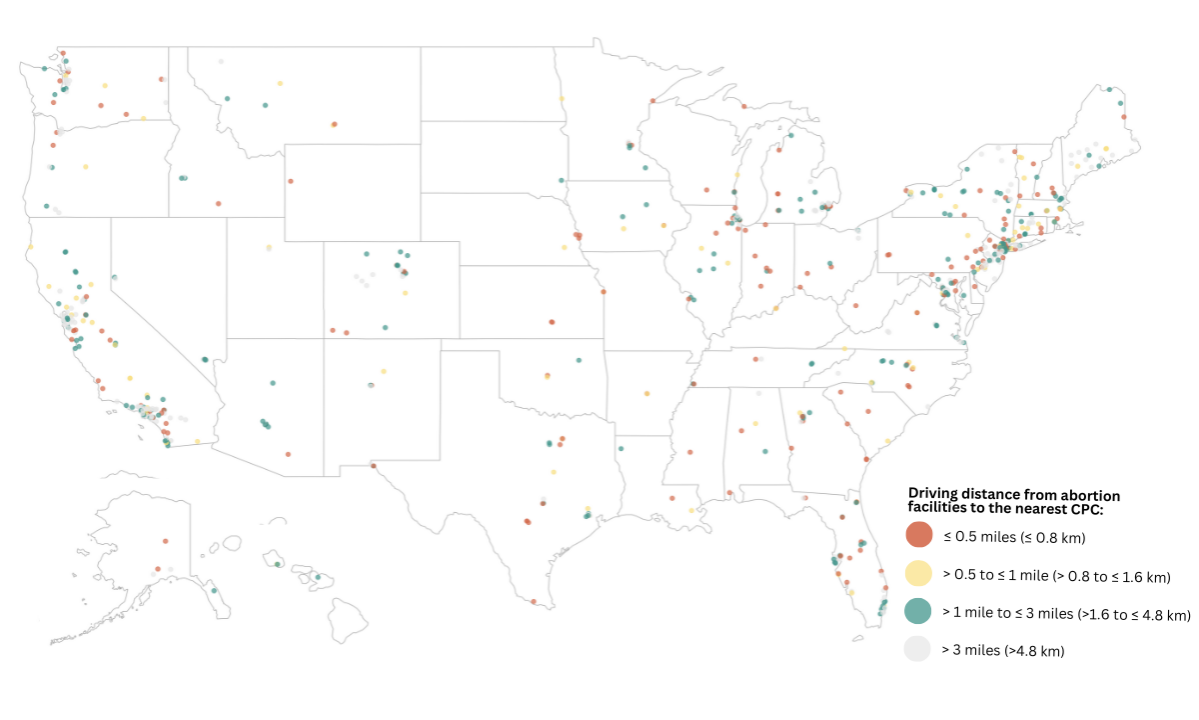
Locations of abortion facilities based on driving distance to the nearest crisis pregnancy center (CPC), United States 2021. Data sources: CPC Map; Advancing New Standards in Reproductive Health Abortion Facility Database.

We calculated the minimum, mean, median, and maximum driving distances (Table 4) and drive times (Table 5) from abortion facilities to the nearest CPC, nationally and by region, division, and state. We also calculated the number and percentage of abortion facilities for which the nearest CPC was within 0.25, 0.5, 1, and 3 driving miles (Table 4) and mapped the locations of abortion facilities based on driving time to the nearest CPC (Figure 3). Nationally, the minimum driving distance was 0.001 miles (approximately 5 feet [1 foot is approximately 30.5 cm]) and the maximum was 119.9 miles. The mean was 3.8 miles and median 2.1 miles. The nearest CPC was located within 0.25 miles of approximately 1 in 7 abortion facilities, within 0.5 miles of approximately one-quarter, within 1 mile for more than one-third, and within 3 miles for two-thirds of abortion facilities in the United States. The minimum drive time was 0.004 minutes (<1 second) and the maximum 122.1 minutes, with a mean of 7.8 minutes and median of 5.5 minutes.

In each of the 4 regions, minimum driving distances were <0.02 miles and minimum drive times were <0.05 minutes or <3 seconds. Median driving distances ranged from 1.6 to 2.7 miles. Maximum driving distances ranged from 7.2 to 119.9 miles. The percentage of abortion facilities with a CPC located within 0.25 and 3 driving miles ranged from 9.6% and 55.1% in the West to 22.4% and 76.5% in the Midwest, respectively. Median drive times ranged from 3.9 to 6.8 minutes, and maximum drive times ranged from 17.2 to 122.1 minutes.

By division, minimum driving distances were <0.1 miles, and minimum drive times were <0.4 minutes or <24 seconds. Median driving distances were <1 mile in 3 divisions and <2.5 miles in all divisions except the Pacific. Maximum driving distances ranged from <10 miles in 5 divisions to >90 miles in 2. The percentage of abortion facilities with a CPC located within 0.25 driving miles ranged from 7.4% to 34.8%. The percentage with a CPC within 3 miles ranged from 51.6% to 95.7%. Median drive times ranged from 2.1 to 7.3 minutes. Maximum drive times ranged from 9.9 to 122.1 minutes.

By state, minimum driving distances from abortion facilities to the nearest CPC were <0.01 miles or <52.8 feet in 4 states. Only DC and 5 states had minimum driving distances >0.5 miles. The greatest minimum driving distance was 2.4 miles. Median driving distances ranged from 0.07 to 10.8 miles. Median driving distances were <0.5 miles in 11 states and >3.0 miles in 2 states. Maximum driving distances were <1.0 mile in 10 states and >13.0 miles in 11 states. Minimum drive times from abortion facilities to the nearest CPC ranged from 0.01 minutes or <1 second to 6.3 minutes. Minimum drive times were >1.0 minute in only 13 states and DC. Median drive times ranged from 0.3 to 19.1 minutes. Median drive times were <2.0 minutes in 11 states. Median drive times were >9 minutes in only 2 states and DC. Maximum drive times ranged from 0.9 to 122.1 minutes. Maximum drive times were <5.0 minutes in 14 states, between 5.0 and <15.0 minutes in 21 states and DC, between 15 and 30 minutes in 10 states, and >30 minutes in 5 states.

The percentage of abortion facilities for which the nearest CPC was located within 0.25 miles ranged from 0% in 14 states to 100% in a single state. The percentage for which the nearest CPC was located within 0.5 miles ranged from 0% in 5 states and DC to 100% in 4 states. The percentage with a CPC within 1 mile ranged from 0% in 1 state and DC to 100% in 9 states. All states and DC had at least 1 abortion facility with a CPC located within 3 miles, and the nearest CPC was located within 3 miles of 100% of abortion facilities in 20 states.

**Table 4:** Driving distance in miles (1 mile is approximately 1.609 km) from abortion facilities to the nearest crisis pregnancy center (CPC) in the United States, 2021.

			Driving distance to nearest CPC (miles)									Driving distance to nearest CPC, n (%)						
			Minimum		Median (IQR)		Mean (SD)		Maximum		≤0.25 miles			≤0.50 miles		≤1 mile		≤3 miles
**United States**			0.001		2.069 (0.597-4.171)		3.812 (8.393)		119.936		107 (14.1)			171 (22.6)		273 (36.1)		502 (66.3)
**Region**																		
	Midwest	0.015		1.582 (0.294-2.894)		1.952 (1.967)		7.239		22 (22.4)			36 (36.7)		45 (45.9)		75 (76.5)	
	Northeast	0.001		1.660 (0.631-3.931)		5.243 (11.806)		90.758		24 (11.3)			42 (19.8)		82 (38.7)		149 (70.3)	
	South	0.004		1.697 (0.388-3.213)		2.102 (1.957)		9.286		35 (20)			51 (29.1)		72 (41.1)		128 (73.1)	
	West	0.009		2.745 (0.949-5.358)		4.467 (8.886)		119.936		26 (9.6)			42 (15.4)		74 (27.2)		150 (55.1)	
**Division**																		
	East North Central	0.015		1.854 (0.316-3.361)		2.219 (2.092)		7.239		14 (18.7)			25 (33.3)		29 (38.7)		53 (70.7)	
	East South Central	0.053		0.956 (0.331-2.391)		1.676 (2.075)		7.246		3 (20)			6 (40)		9 (60)		13 (86.7)	
	Middle Atlantic	0.001		1.782 (0.622-3.931)		4.064 (8.326)		59.742		19 (13.2)			30 (20.8)		53 (36.8)		100 (69.4)	
	Mountain	0.085		2.347 (0.935-3.948)		4.153 (7.525)		40.578		6 (10.2)			11 (18.6)		16 (27.1)		40 (67.8)	
	New England	0.060		1.240 (0.822-3.396)		7.741 (16.783)		90.758		5 (7.4)			12 (17.6)		29 (42.6)		49 (72.1)	
	Pacific	0.009		2.845 (0.961-5.541)		4.554 (9.242)		119.936		20 (9.4)			31 (14.6)		58 (27.2)		110 (51.6)	
	South Atlantic	0.004		2.221 (0.397-3.512)		2.293 (1.976)		9.286		27 (20.8)			34 (26.2)		45 (34.6)		88 (67.7)	
	West North Central	0.039		0.554 (0.257-1.837)		1.082 (1.138)		3.804		8 (34.8)			11 (47.8)		16 (69.6)		22 (95.7)	
	West South Central	0.035		0.828 (0.397-2.674)		1.488 (1.703)		7.997		5 (16.7)			11 (36.7)		18 (60)		27 (90)	
**State**																		
	Alabama	0.092		0.966 (0.390-2.545)		1.839 (2.104)		5.199		1 (20)			2 (40)		3 (60)		4 (80)	
	Alaska	0.194		2.520 (0.580-10.701)		26.786 (52.246)		119.936		1 (20)			2 (40)		2 (40)		3 (60)	
	Arizona	0.184		1.896 (1.061-2.227)		1.693 (1.070)		3.328		0 (0)			2 (25)		2 (25)		7 (87.5)	
	Arkansas	0.049		0.483 (0.266-0.700)		0.483 (0.613)		0.917		1 (50)			1 (50)		2 (100)		2 (100)	
	California	0.027		2.987 (1.071-5.373)		3.946 (4.798)		50.663		13 (7.9)			20 (12.2)		42 (25.6)		83 (50.6)	
	Colorado	0.085		2.744 (1.586-4.913)		6.633 (11.070)		40.578		3 (12.5)			4 (16.7)		5 (20.8)		14 (58.3)	
	Connecticut	0.083		1.049 (0.834-2.185)		2.169 (2.598)		8.717		2 (12.5)			3 (18.8)		8 (50)		12 (75)	
	Delaware	0.042		0.869 (0.455-1.283)		0.869 (1.170)		1.697		1 (50)			1 (50)		1 (50)		2 (100)	
	District of Columbia	1.027		1.890 (1.479-2.445)		2.034 (0.978)		3.329		0 (0)			0 (0)		0 (0)		3 (75)	
	Florida	0.004		2.586 (0.643-3.447)		2.405 (1.787)		5.735		10 (18.2)			13 (23.6)		17 (30.9)		34 (61.8)	
	Georgia	0.035		1.090 (0.095-2.497)		1.938 (2.330)		7.411		6 (42.9)			6 (42.9)		7 (50)		11 (78.6)	
	Hawaii	0.833		1.953 (1.393-2.309)		1.817 (0.924)		2.666		0 (0)			0 (0)		1 (33.3)		3 (100)	
	Idaho	0.433		2.109 (1.147-3.527)		2.565 (2.258)		5.612		0 (0)			1 (25)		1 (25)		3 (75)	
	Illinois	0.076		1.854 (0.726-3.018)		2.205 (1.777)		5.901		1 (3.7)			6 (22.2)		9 (33.3)		20 (74.1)	
	Indiana	0.028		0.290 (0.102-0.319)		0.451 (0.694)		2.000		3 (42.9)			6 (85.7)		6 (85.7)		7 (100)	
	Iowa	0.041		0.853 (0.526-1.397)		1.072 (0.937)		2.688		1 (16.7)			2 (33.3)		4 (66.7)		6 (100)	
	Kansas	0.070		0.318 (0.126-0.507)		0.315 (0.244)		0.554		2 (50)			3 (75)		4 (100)		4 (100)	
	Kentucky	0.272		0.438 (0.355-0.520)		0.438 (0.234)		0.603		0 (0)			1 (50)		2 (100)		2 (100)	
	Louisiana	0.148		0.694 (0.421-1.091)		0.777 (0.674)		1.488		1 (33.3)			1 (33.3)		2 (66.7)		3 (100)	
	Maine	0.394		10.826 (1.769-28.158)		20.857 (26.599)		90.758		0 (0)			1 (5)		4 (20)		8 (40)	
	Maryland	0.029		2.218 (0.565-4.367)		2.574 (2.396)		9.286		4 (20)			5 (25)		5 (25)		13 (65)	
	Massachusetts	0.060		1.309 (0.843-2.658)		2.709 (3.909)		13.499		1 (5.6)			3 (16.7)		8 (44.4)		16 (88.9)	
	Michigan	0.015		2.470 (0.360-4.110)		2.804 (2.411)		7.239		6 (21.4)			8 (28.6)		8 (28.6)		17 (60.7)	
	Minnesota	0.069		2.137 (0.257-2.915)		1.765 (1.552)		3.804		3 (42.9)			3 (42.9)		3 (42.9)		6 (85.7)	
	Mississippi	0.070		0.070 (0.070-0.070)		0.070 (—^a^)		0.070		1 (100)			1 (100)		1 (100)		1 (100)	
	Missouri	0.471		0.471 (0.471-0.471)		0.471 (—)		0.471		0 (0)			1 (100)		1 (100)		1 (100)	
	Montana	0.205		1.750 (0.668-2.733)		3.429 (5.048)		13.501		1 (16.7)			1 (16.7)		3 (50)		5 (83.3)	
	Nebraska	0.039		0.425 (0.232-0.668)		0.459 (0.437)		0.912		2 (66.7)			2 (66.7)		3 (100)		3 (100)	
	Nevada	0.097		1.951 (1.430-3.938)		2.645 (1.983)		6.670		1 (11.1)			1 (11.1)		1 (11.1)		6 (66.7)	
	New Hampshire	0.069		1.273 (0.359-2.229)		3.340 (5.704)		14.825		1 (16.7)			3 (50)		3 (50)		5 (83.3)	
	New Jersey	0.002		2.227 (0.955-4.992)		3.458 (3.319)		13.373		2 (4.9)			8 (19.5)		11 (26.8)		24 (58.5)	
	New Mexico	0.137		2.979 (0.961-3.446)		2.296 (1.658)		3.957		1 (20)			1 (20)		2 (40)		3 (60)	
	New York	0.001		1.974 (0.641-3.653)		4.831 (10.355)		59.742		11 (12.6)			14 (16.1)		30 (34.5)		62 (71.3)	
	North Carolina	0.158		1.457 (0.348-3.079)		2.163 (2.272)		8.259		3 (18.8)			4 (25)		7 (43.8)		12 (75)	
	North Dakota	0.610		0.610 (0.610-0.610)		0.610 (—)		0.610		0 (0)			0 (0)		1 (100)		1 (100)	
	Ohio	0.046		2.718 (0.192-3.687)		2.498 (2.206)		6.702		3 (33.3)			3 (33.3)		3 (33.3)		5 (55.6)	
	Oklahoma	0.466		0.582 (0.524-1.631)		1.242 (1.246)		2.680		0 (0)			1 (33.3)		2 (66.7)		3 (100)	
	Oregon	0.015		4.094 (1.806-5.712)		4.418 (4.326)		16.805		1 (7.7)			2 (15.4)		3 (23.1)		5 (38.5)	
	Pennsylvania	0.081		0.473 (0.212-1.318)		1.445 (2.247)		7.779		6 (37.5)			8 (50)		12 (75)		14 (87.5)	
	Rhode Island	0.925		1.061 (0.993-1.129)		1.061 (0.193)		1.197		0 (0)			0 (0)		1 (50)		2 (100)	
	South Carolina	0.024		0.166 (0.095-0.325)		0.224 (0.235)		0.483		2 (66.7)			2 (66.7)		3 (100)		3 (100)	
	South Dakota	2.387		2.387 (2.387-2.387)		2.387 (—)		2.387		0 (0)			0 (0)		0 (0)		1 (100)	
	Tennessee	0.053		1.507 (0.707-2.391)		2.143 (2.422)		7.246		1 (14.3)			2 (28.6)		3 (42.9)		6 (85.7)	
	Texas	0.035		0.899 (0.397-2.751)		1.710 (1.889)		7.997		3 (13.6)			8 (36.4)		12 (54.5)		19 (86.4)	
	Utah	0.858		2.927 (1.892-3.961)		2.927 (2.926)		4.996		0 (0)			0 (0)		1 (50)		1 (50)	
	Vermont	0.134		0.550 (0.414-0.797)		0.605 (0.358)		1.146		1 (16.7)			2 (33.3)		5 (83.3)		6 (100)	
	Virginia	0.222		2.723 (1.576-4.064)		2.779 (1.707)		5.794		1 (6.7)			2 (13.3)		4 (26.7)		9 (60)	
	Washington	0.009		1.689 (0.690-8.578)		4.503 (4.931)		15.836		5 (17.9)			7 (25)		10 (35.7)		16 (57.1)	
	West Virginia	0.351		0.351 (0.351-0.351)		0.351 (—)		0.351		0 (0)			1 (100)		1 (100)		1 (100)	
	Wisconsin	0.052		0.522 (0.305-0.897)		0.681 (0.677)		1.626		1 (25)			2 (50)		3 (75)		4 (100)	
	Wyoming	0.339		0.339 (0.339-0.339)		0.339 (—)		0.339		0 (0)			1 (100)		1 (100)		1 (100)	

^a^Not applicable.

**Table 5:** Driving times in minutes from abortion facilities to the nearest crisis pregnancy center (CPC) in the United States, 2021.

		Drive time to the nearest CPC (minutes)			
		Minimum	Median (IQR)	Mean (SD)	Maximum
**United States**		0.004	5.528 (2.179-10.159)	7.772 (10.733)	122.058
**Region**					
	Midwest	0.049	3.859 (1.123-7.090)	4.762 (4.234)	17.508
	Northeast	0.004	5.776 (2.609-10.698)	10.113 (15.430)	117.400
	South	0.037	4.607 (1.285-7.668)	5.062 (4.092)	17.155
	West	0.035	6.816 (3.074-11.860)	8.775 (10.272)	122.058
**Division**					
	East North Central	0.049	4.716 (1.104-7.915)	5.262 (4.490)	17.508
	East South Central	0.262	2.669 (1.464-5.937)	4.176 (3.997)	14.319
	Middle Atlantic	0.004	6.242 (2.886-11.044)	8.852 (10.935)	78.524
	Mountain	0.390	5.566 (2.793-8.538)	7.482 (8.605)	48.780
	New England	0.285	4.517 (2.549-10.097)	12.784 (21.999)	117.400
	Pacific	0.035	7.291 (3.092-12.261)	9.133 (10.679)	122.058
	South Atlantic	0.037	5.491 (1.314-8.448)	5.548 (4.218)	17.155
	West North Central	0.089	2.139 (1.214-5.227)	3.133 (2.758)	9.886
	West South Central	0.174	2.416 (1.113-4.988)	3.398 (3.045)	10.895
**State**					
	Alabama	0.406	4.123 (1.342-6.354)	4.556 (4.091)	10.554
	Alaska	0.702	4.625 (1.790-15.605)	28.956 (52.380)	122.058
	Arizona	1.145	4.261 (2.663-5.038)	3.935 (2.052)	7.051
	Arkansas	0.316	1.446 (0.881-2.011)	1.446 (1.597)	2.575
	California	0.050	7.460 (3.261-11.923)	8.578 (7.249)	67.039
	Colorado	0.684	6.499 (4.675-11.007)	10.438 (12.172)	48.780
	Connecticut	0.364	3.559 (2.586-8.126)	5.283 (4.190)	12.630
	Delaware	0.152	3.814 (1.983-5.644)	3.814 (5.178)	7.475
	District of Columbia	5.340	9.547 (7.718-11.091)	9.263 (3.106)	12.617
	Florida	0.037	6.108 (1.757-8.332)	5.576 (3.784)	13.998
	Georgia	0.212	2.507 (0.637-6.744)	4.533 (4.725)	14.283
	Hawaii	2.642	5.982 (4.312-6.717)	5.358 (2.465)	7.451
	Idaho	1.628	4.917 (3.398-7.784)	6.266 (5.186)	13.601
	Illinois	0.557	4.977 (2.369-8.803)	6.027 (4.612)	17.508
	Indiana	0.049	0.984 (0.551-1.292)	1.335 (1.534)	4.623
	Iowa	0.277	2.417 (2.117-3.789)	3.051 (2.273)	6.930
	Kansas	0.594	1.177 (0.898-1.436)	1.157 (0.467)	1.680
	Kentucky	1.586	1.935 (1.761-2.109)	1.935 (0.493)	2.284
	Louisiana	0.825	2.722 (1.773-3.066)	2.319 (1.339)	3.410
	Maine	1.087	19.075 (5.035-41.101)	30.216 (34.250)	117.400
	Maryland	0.155	5.268 (1.890-9.885)	6.180 (4.994)	15.795
	Massachusetts	0.285	4.402 (3.364-6.959)	6.818 (7.186)	27.511
	Michigan	0.086	5.794 (1.129-8.310)	5.829 (4.708)	15.880
	Minnesota	0.442	6.308 (1.214-7.171)	4.772 (3.747)	9.886
	Mississippi	0.320	0.320 (1.761-2.109)	0.320 (0.493)	0.320
	Missouri	2.400	2.400 (2.400-2.400)	2.400 (—^a^)	2.400
	Montana	0.390	4.984 (1.530-7.537)	6.074 (6.314)	17.317
	Nebraska	0.089	2.139 (1.114-2.498)	1.695 (1.436)	2.857
	Nevada	0.860	5.807 (4.402-8.337)	6.414 (3.573)	12.101
	New Hampshire	0.363	3.873 (1.690-6.478)	6.108 (7.371)	20.231
	New Jersey	0.010	7.031 (3.532-11.872)	7.997 (5.344)	20.127
	New Mexico	1.033	6.087 (2.394-8.465)	5.351 (3.513)	8.776
	New York	0.004	6.523 (3.453-11.805)	10.127 (13.271)	78.524
	North Carolina	0.227	4.217 (1.716-8.939)	5.403 (4.893)	17.155
	North Dakota	1.960	1.960 (1.960-1.960)	1.960 (—)	1.960
	Ohio	0.252	7.093 (0.548-8.266)	5.550 (4.211)	11.279
	Oklahoma	1.264	1.633 (1.448-4.155)	3.191 (3.024)	6.676
	Oregon	0.064	9.347 (5.137-13.664)	9.768 (6.578)	22.432
	Pennsylvania	0.526	2.540 (0.936-5.178)	4.112 (4.313)	13.736
	Rhode Island	3.798	4.207 (4.003-4.412)	4.207 (0.578)	4.616
	South Carolina	0.232	0.718 (0.475-0.792)	0.605 (0.331)	0.865
	South Dakota	6.276	6.276 (6.276-6.276)	6.276 (—)	6.276
	Tennessee	0.262	4.366 (2.424-5.937)	5.096 (4.564)	14.319
	Texas	0.174	2.416 (1.204-5.850)	3.751 (3.313)	10.895
	Utah	3.084	6.224 (4.654-7.793)	6.224 (4.440)	9.363
	Vermont	0.658	2.144 (1.487-2.519)	2.111 (1.086)	3.808
	Virginia	0.889	7.023 (3.551-8.619)	6.192 (3.419)	11.198
	Washington	0.035	4.499 (2.192-14.800)	8.957 (8.692)	26.951
	West Virginia	1.615	1.615 (1.615-1.615)	1.615 (—)	1.615
	Wisconsin	0.165	1.749 (1.109-2.988)	2.348 (2.390)	5.730
	Wyoming	0.983	0.983 (0.983-0.983)	0.983 (—)	0.983

^a^Not applicable.

## Discussion

### Principal Findings

This study aimed to examine the geographic landscape of CPCs and abortion facilities in the United States in 2021 and is the first to directly examine CPC locations relative to abortion facilities. We found that the ratio of CPCs to abortion facilities in the United States was 3.4 in 2021, similar to the ratio of 3.2 that we reported using the same data sets in 2018 [4]. The number of CPCs was similar in 2018 (before COVID-19) and 2021 (during the active COVID-19 pandemic). Although new centers opened after 2018 and before the pandemic and some CPCs benefited from federal government support programs during the pandemic, such as the Paycheck Protection Program [49], and continued to operate during the early years of the pandemic, others closed [50]. The extent to which CPCs reopened after the height of the pandemic is currently being studied. Consistent with our 2018 results [4], ratios of CPCs to abortion facilities were highest in the South and Midwest regions and the West North Central, East South Central, and West South Central divisions. Similarly, Missouri, Kentucky, Mississippi, and Wisconsin had the highest ratios and California, DC, Maine, Nevada, and New Jersey had the lowest ratios in both 2018 [4] and 2021. In our 2018 analyses [4], we reported that an increasing number of CPCs per state was associated with an increased likelihood of the introduction of legislation to ban all or most abortions [4]. Notably, abortion is currently completely banned in all 6 states where the ratio of CPCs to abortion facilities was >15.0 in 2021. In addition, the ratio was >7.0 in 13 of the 14 states that currently completely ban abortion.

Nationally, more than half (131,410/239,462, 55%) of block groups were within 15 miles of a CPC and an abortion facility. More than one-quarter (63,679/239,462, 27%) were categorized in the “CPC only” zone, meaning that 82% (195,089/239,462) of all block groups in the United States were located within 15 miles of a CPC in 2021. The percentage of block groups within 15 miles of both a CPC and an abortion facility was highest (at least 70%) in the Northeast and West regions and the Middle Atlantic and Pacific divisions, where the number of abortion facilities was highest. Although approximately two-thirds (484/757, 64%) of all abortion facilities nationally were located in the Northeast and West regions and approximately half (375/757, 47%) in the Middle Atlantic and Pacific divisions [51], <3% block groups in each of these areas were categorized as “abortion facility only” zones. Differences by region and division may be due to differences in urbanicity and population centers. In all regions, divisions, and all but 5 states there were more block groups within a 15-mile radius of a CPC than outside of that distance. In 20 states and DC, >50% of block groups were categorized as being within 15 miles of both a CPC and an abortion facility (“dual presence” zone).

Nationally, 60% of women of reproductive age, 45.2 million women, lived within 15 miles of both a CPC and an abortion facility, and 26%, approximately 19.7 million, lived within 15 miles of a CPC only. Less than 15% of women of reproductive age in the United States lived >15 miles from a CPC. In contrast, less than one-third lived within 15 miles of an abortion facility, and <1%, approximately 625,000 women, lived within 15 miles of an abortion facility only. In all regions, divisions, and states, except North Dakota, more women lived within a 15-mile radius of a CPC than outside of that distance.

CPCs were located exceptionally close to abortion facilities in the United States in 2021. In all regions, divisions, and 34 states, the minimum distance from an abortion facility to the nearest CPC was <0.1 miles or <528 feet. The median distance was 2 miles nationally and <2 miles in all regions except the West, all but 3 divisions, and 33 states and DC. Median drive time to the nearest CPC was 5.5 minutes nationally and <5 miles in 2 of 4 regions, all but 4 divisions, and 31 states. Nationally, the nearest CPC was within 3 miles of most abortion facilities and the nearest CPC was within 0.5 miles of approximately one-quarter (171/757, 22.6%) of all abortion facilities.

### Limitations

This study is subject to several limitations. In general, spatial analyses that use the smallest feasible geographic unit available better limit bias and increase precision. This study used block groups for feasibility reasons. Although census blocks are the smallest geographic unit for which age and sex data were available, such analyses are computationally- and resource-intensive at the national level. Block groups may result in small underestimates of abortion accessibility relative to census blocks but are much more precise than county-level analyses and facilitated examination of the number and percentage of women of reproductive age living in each zone in this study [47]. Further, selection of different driving distances to define each zone and catchment area of reproductive-aged women could influence the findings. We opted to define buffer zones based on a 15-mile radius from CPCs and abortion facilities based on prior research on median distances to abortion facilities nationally in the United States and given the relative number of CPCs as compared with abortion facilities. In addition, misclassification of CPCs and abortion facilities could be possible given that the 2 national data sources did not continuously collect data in 2021. Finally, this study does not account for density of CPCs and abortion facilities, number of staff or volunteers, types of services offered, volume and types of or potential targets of advertising, or other factors that may influence individual decision-making about health-seeking. Despite limitations, the study offers significant strengths, including use of scientifically rigorous national data sources; analyses unrestricted to state boundaries in line with behaviors of people seeking health services; advanced spatial methods that produced estimates of driving distances and drive times; and novel approaches to examine an understudied health topic of critical importance.

### Comparison With Prior Work

Although other researchers have examined geographical access to abortion facilities in the United States [12,16], distances patients travel to obtain abortion care nationally [48], and compared categorizations of drive times to abortion facilities and CPCs [52], this is the first study to directly examine relative geographic access to CPCs and abortion facilities using the lens of CPCs’ long-standing geographic tactic of locating near abortion facilities. In addition, this is the first published study to report the proximity of CPCs to abortion facilities nationally.

Only one previous study has examined populations in proximity to CPCs and abortion facilities [52]. The study used a CPC database that included shelters, thrift shops, adoption agencies, and administrative offices that did not offer pregnancy testing and information designed to influence pregnancy options toward childbirth. The study also compared relative distances to the nearest CPC and abortion facility based on 30- and 60-mile drive time categorizations for the total US population, including all genders, children, and older adults. This study used a public health approach, concentrating on centers that most directly aimed to attract people seeking or considering abortion care; it was limited to antiabortion “pregnancy centers” that offered free pregnancy testing (eg, excluded thrift stores and administration offices) and excluded adoption agencies and maternity homes [52]. Thus, findings from this study are presumed to be both more precise and conservative than previous research.

### Conclusions

Despite their risks to individual, family, and public health, this study’s findings suggest that, before the *Dobbs* decision, CPCs’ tactic of locating near abortion facilities was largely realized, and the centers were “positioned” to attempt to intercept people considering and seeking abortion, given their relative numbers and locations, and close proximity to abortion facilities. Estimates have likely changed drastically since the *Dobbs* decision as some states banned or severely restricted abortion and increased funding for CPCs and other states have passed protections and expanded abortion access. The number and locations of CPCs after the *Dobbs* decision are currently being studied. It is possible but remains to be seen if CPCs proliferated in the states that increased funding for the centers and proliferated in states where abortion remains legal and protected.

Research shows that many people seek sources of sexual and reproductive health care on the internet, including abortion [53]. Most (>96%) CPCs advertise to potential clients via websites [54]. Currently, some states direct people seeking abortion to CPCs through mandated counseling. The federal government provides referrals to CPCs through its web-based HIV and sexually transmitted infection service locators powered by the Centers for Disease Control and Prevention’s contract with the National Prevention Information Network [55]. Given CPCs’ health risk and that most women in the United States reside within 15 miles of a CPC, location-aware digital tools, such as Yelp, which currently identifies CPCs, and tools with location-based filters, such as CPC Map [4], created with a primary goal of helping people seeking health services identify CPCs, can be valuable for assisting people seeking safe, evidence-based sexual and reproductive health care identify and avoid CPCs. In addition, public health and medical professionals and advocates should make themselves aware of CPCs operating in their areas and help educate the public and patients about CPCs and their potential harms and where to find safe, quality sources of care and information [5]. Tailored programming to raise awareness about CPCs based on geography may help people avoid health care delays and adverse outcomes.

CPCs continue to operate largely unregulated [6]. Identical federal legislation to regulate CPCs from providing inaccurate health information and engaging in deceptive advertising was introduced into the House and Senate in 2022 but has not been passed [55,56]. Efforts to regulate CPCs at the state level have not been entirely successful. For example, a law passed in California to require CPCs post signage was overturned by the United States Supreme Court, and the state of Illinois agreed not to enforce a law that imposed fines for CPCs providing misleading health information after a federal judge ruled in favor of CPCs’ First Amendment free speech rights [57,58]. Connecticut in 2021 and Vermont in 2023 passed laws barring CPCs from engaging in false and misleading advertising [30,38]. Some cities have passed local ordinances to regulate CPCs and to prevent them from locating in their areas and have made zoning decisions to prevent CPCs from locating near abortion facilities [59-61].

Given few currently available regulatory strategies and increased funding for CPCs in many states since *Dobbs*, to minimize harm, public health and medical professionals, advocates, researchers, funders, and government officials should prioritize: (1) raising awareness about CPCs, including awareness about local CPCs and safe sources of health care; (2) urging governments to refrain from supporting, referring to, and funding CPCs; (3) urging government regulation of CPCs; and (4) identifying CPCs’ strategies and tactics, especially after the *Dobbs* decision—in addition to facilitating, providing, and advocating for safe, respectful, accessible, appropriate, and effective sexual and reproductive health care for all.

## Abbreviations

ANSIRH: Advancing New Standards in Reproductive Health
CPC: crisis pregnancy center
DC: District of Columbia
